# Nanotitanium dioxide toxicity in mouse lung is reduced in sanding dust from paint

**DOI:** 10.1186/1743-8977-9-4

**Published:** 2012-02-02

**Authors:** Anne Thoustrup Saber, Nicklas Raun Jacobsen, Alicia Mortensen, Józef Szarek, Petra Jackson, Anne Mette Madsen, Keld Alstrup Jensen, Ismo K Koponen, Gunnar Brunborg, Kristine Bjerve Gützkow, Ulla Vogel, Håkan Wallin

**Affiliations:** 1 The National Research Centre for the Working Environment, Lersø Parkallé 105, DK-2100 Copenhagen, Denmark; 2 National Food Institute, Technical University of Denmark, Mørkhøj Bygade 19, DK-2860 Søborg, Denmark; 3 University of Warmia and Mazury, Faculty of Veterinary Medicine, 10-719 Olsztyn, Poland; 4 Department of Micro and Nanotechnology, Technical University of Denmark, DK-2800 Lyngby, Denmark; 5 Institute of Public Health, University of Copenhagen, DK-1014 Copenhagen K, Denmark; 6 Department of Chemical Toxicology, Division of Environmental Medicine, Norwegian Institute of Public Health, PO Box 4404 Nydalen, N-0403 Oslo, Norway

**Keywords:** Nanoparticles, Nano titanium dioxide, UV-Titan L181, sanding dusts, paint matrix, inflammation, DNA damage, liver histology

## Abstract

**Background:**

Little is known of how the toxicity of nanoparticles is affected by the incorporation in complex matrices. We compared the toxic effects of the titanium dioxide nanoparticle UV-Titan L181 (NanoTiO_2_), pure or embedded in a paint matrix. We also compared the effects of the same paint with and without NanoTiO_2_.

**Methods:**

Mice received a single intratracheal instillation of 18, 54 and 162 μg of NanoTiO_2 _or 54, 162 and 486 μg of the sanding dust from paint with and without NanoTiO_2_. DNA damage in broncheoalveolar lavage cells and liver, lung inflammation and liver histology were evaluated 1, 3 and 28 days after intratracheal instillation. Printex 90 was included as positive control.

**Results:**

There was no additive effect of adding NanoTiO_2 _to paints: Therefore the toxicity of NanoTiO_2 _was reduced by inclusion into a paint matrix. NanoTiO_2 _induced inflammation in mice with severity similar to Printex 90. The inflammatory response of NanoTiO_2 _and Printex 90 correlated with the instilled surface area. None of the materials, except of Printex 90, induced DNA damage in lung lining fluid cells. The highest dose of NanoTiO_2 _caused DNA damage in hepatic tissue 1 day after intratracheal instillation. Exposure of mice to the dust from paints with and without TiO_2 _was not associated with hepatic histopathological changes. Exposure to NanoTiO_2 _or to Printex 90 caused slight histopathological changes in the liver in some of the mice at different time points.

**Conclusions:**

Pulmonary inflammation and DNA damage and hepatic histopathology were not changed in mice instilled with sanding dust from NanoTiO_2 _paint compared to paint without NanoTiO_2_. However, pure NanoTiO_2 _caused greater inflammation than NanoTiO_2 _embedded in the paint matrix.

## Background

Paints and lacquers represent a product group in which nanomaterials are increasingly used due to improvement of characteristics of the products [1]. One example is the addition of nanosized titaniumdioxide (TiO_2_) to paints [1]. Whereas bulk sized TiO_2 _has been used for decades as a whitening agent in paints, nanosized TiO_2 _is added to paints for example as UV-filters or to improve their rheology, for providing self-cleaning properties or for removing odours from air [1]. It is estimated that the global use of TiO_2 _for paint and surface applications exceeds 2.5 million tonnes annually (2005) [2].

Some TiO_2 _nanoparticles have been shown to have inflammogenic, oxidative, and genotoxic effects (reviewed in [3]). Recently, IARC classified TiO_2 _as possibly carcinogenic to humans (Group 2B). This was based on an evaluation showing sufficient evidence of carcinogenicity in experimental animals and inadequate evidence for human carcinogenicity [2].

Whereas exposure may occur during handling and use of freely dispersed nanomaterials [4,5], very little is known of the emissions of nanomaterials during the life cycle (e.g., finishing, sanding, abrasion, weathering and incineration) of products containing nanomaterials [6-10]. There are also much more data on the adverse effects of pure nanomaterials [11], but almost nothing is known on the toxicity of nanoparticles incorporporated into matrices such as paints and plastics [6,12,13].

We have previously characterised the dust obtained from sanding wooden boards painted with different types of paints or lacquers with and without nanoparticles [7]. Sanding generated both nano- and micrometer-sized particles. Sanding of some of the nanoparticle containing paints led to increased formation of nanosized particles compared to the reference paint, but there was no consistent pattern in which paints gave rise to increased formation of nanosized sanding particles, nor the amount [7]. We have screened the toxicity of the pure nanomaterials [14] and of the sanding dust in mice using a single dose (54 μg) and a single time point after intratracheal instillation (1 day) [12].

Based on this screening [12,14], we selected sanding dusts from a conventional indoor paint with and without NanoTiO_2 _(Indoor-NanoTiO_2 _and Indoor-R, respectively) and the same nanosized TiO_2 _as the one included in the paint (NanoTiO_2_, UV-Titan L181) which induced inflammation and DNA damage. The purpose of the present study was to test the toxicity, by inflammatory and DNA damaging effects, of the materials for dose-responses at different time points. The carbon black type Printex 90 was included as a positive control because we have previously shown that Printex 90 is a potent ROS producer [15] that causes both inflammation, DNA strand breaks and mutations [16].

We evaluated the inflammatory response by characterizing the presence of inflammatory cells in lung fluids. The fibrotic response was analysed by measuring mRNA expression of *Tgf-beta *because some nanomaterials have been shown to induce fibrosis [17]. DNA damage was analysed by measuring DNA strand breaks in broncheoalveolar lavage cells and liver tissue by the Comet assay as a sensitive assay for genotoxicity.

## Results

### Physicochemical characterization of particles

The Danish paint and lacquer industry provided an indoor acrylic paint product with 10% content of NanoTiO_2 _(referred as Indoor-NanoTiO_2_) and a corresponding product without nanomaterials (referred to as Indoor R) (Table 1). The tested pure nanomaterials included a nanosized TiO_2 _material (UV-Titan L181, code: NanoTiO_2_), and carbon black (Printex 90), which was used as an internal reference particle. Physicochemical characteristics of the particles and the dusts used in the study are presented in detail in our three recent publications [7,12,14].

**Table 1:** Composition of paints^a^

	Type of TiO_2_	Indoor-R^b^	Indoor-NanoTiO_2_^c^
Water		15	15
Natrosol 250 HR		0.23	0.23
Dispex 40		0.56	0.56
Proxel B D 20		0.23	0.23
Propylenglycole		6.8	6.8
UV-Titan L181	NanoTiO_2_	0	10
Tronox R-KB-2	PigmentTiO_2_	23.8	13.8
Plextol D510		49.8	49.8
Carnauba waxemulsion20%		1.75	1.75
Butyldiglycole		1.31	1.31
Collacral 8503		0.13	0.13
Dehydran 1227		0.17	0.17
Ammonia water 24%		0.22	0.22

Total		100	100

^a^Numbers are given in mass % of total. ^b^Indoor-R: Indoor paint without NanoTiO_2 _^c^Indoor-NanoTiO_2_: Indoor paint with NanoTiO_2_

In brief, the UV-Titan L181 (NanoTiO_2_) was a rutile coated with Si, Al, Zr and polyalcohol [14]. The average crystallite size was determined to be 20.6 nm and the powder had a specific surface area of 107.7 m^2^/g. By electron microscopy of the particle suspensions used for instillation, the primary particles occured in open to dense aggregates of ca. 100 nm or larger. Printex 90 consisted of small to very large aggregates of primary carbonaceous spheres [14]. The specific surface area was on the order of 295-338 m^2^/g. The primary particle size is reported to be 14 nm by the manufacturer, but has a wide size-range. In the dispersion mediums the aggregate size varied from less than 100 nm into the micrometer-range. The typical aggregate size was ca. 200 nm.

The collected sanding dusts (Indoor-R and Indoor-NanoTiO_2_) were in the respirable size fraction [7]. In both cases the airborne dust could be resolved into five size-modes ranging from ca. 10 nm to ca. 1.7 μm. The two smallest modes at ca. 10 and 12.7 nm mainly if not completely originated from the sanding machine. However, as shown in Sharma et al. [18], the collection efficiency of the electrostatic precipitator rapidly decreases for particles smaller than ca. 30 nm reducing the importance of these particles in the sampled test materials. We have reported that the coarse particles were dominated by paint aggregates whereas the smaller particles to greater extent consisted of free to aggregated pigments and smaller paint aggregates as determined by scanning electron microscopy [14].

The endotoxin content in supernatants from particle suspensions was assessed using the *Limulus Amebocyte *lysate enzyme assay (LAL). The endotoxin contents measured in the 4.05 mg/ml particle suspensions were: 0.012 ng/ml (0.19 EU/ml) for NanoTiO_2 _and 0.011 ng/ml (0.17 EU/ml) for Printex 90. The contents of the two dust suspensions were 0.16 ng/ml (2.43 EU/ml) for Indoor-NanoTiO_2 _and 0.024 ng/ml (0.36 EU/ml) for Indoor-R. The amount of endotoxin received by mice given the 162 μg dose was for all the tested particles and dusts below 0.1 EU, a dose equivalent to 6 pg endotoxin, or 0.3 ng endotoxin/kg body weight.

### Cell count in broncho-alveolar lavage fluid

**Figure 1:**
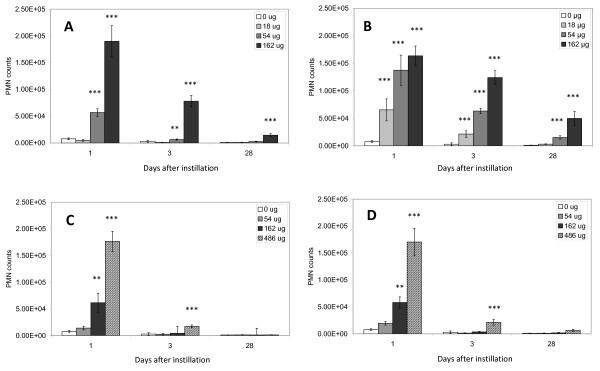
**Neutrophil influx in the lungs**. Neutrophil influx (PMN) in the lungs of mice exposed to 0, 18, 54 or 162 μg of NanoTiO_2 _(A) or Printex 90 (B) or 0, 54, 162 or 486 μg of Indoor-R (C) or Indoor-NanoTiO_2 _(D). *, **, ***: Statistically significant compared to control mice at the 0.5, 0.01 and 0.001 level, respectively.

To assess the recruitment of inflammatory cells into the lung lumen, we determined the total number of BAL cells and the number of macrophages, neutrophils, eosinophils and lymphocytes in the BAL cells (Table 2). The neutrophil influx is also shown in Figure 1. Data from the control group and the Printex 90 exposed mice have been published elsewhere [19].

**Table 2:** BAL fluid counts in mice.

		Control		NanoTiO_2_			Indoor-R			Indoor-NanoTiO_2_	
			
1 day			18 μg	54 μg	162 μg	54 μg	162 μg	486 μg	54 μg	162 μg	486 μg
	Neutrophils (x10^3^)	7.7 ± 1.7	4.9 ± 1.5	56.8 ± 7.2***	189.9 ± 29.1***	14.2 ± 3.5	61.7 ± 17.8**	176.5 ± 18.8***	19.7 ± 3.5	58.2 ± 10.5**	170.4 ± 25.3***
	Macrophages (x10^3^)	53.2 ± 2.5	64.1 ± 6.9	56.4 ± 5.8	41.4 ± 6.2	61.1 ± 7.7	69.5 ± 4.1	78.9 ± 13.1	73.0 ± 7.1	68.8 ± 6.6	51.4 ± 7.2
	Eosinophils (x10^3^)	0.3 ± 0.4	0.3 ± 0.2	1.3 ± 0.7	2.1 ± 0.6	0.2 ± 0.1	2.0 ± 1.0	20.5 ± 3.1***	0.2 ± 0.2	1.3 ± 0.5	10.1 ± 5.4***
	Lymphocytes (x10^3^)	1.6 ± 0.2	1.2 ± 0.5	1.0 ± 0.3	2.4 ± 1.0	0.7 ± 0.2	2.1 ± 0.8	3.4 ± 1.4	0.9 ± 0.2	2.2 ± 0.8	2.7 ± 1.0
	Total BAL cells (x10^3^)	74.0 ± 3.6	77.6 ± 8.8	123 ± 12.8**	246.5 ± 31.1***	85.4 ± 8.2	146.4 ± 22.1***	298.8 ± 25.5***	106.3 ± 9.5	139.2 ± 14.2***	256.3 ± 29.5***
3days											

	Neutrophils (x10^3^)	3.0 ± 2.3	0.9 ± 0.5	6.5 ± 1.0**	78.1 ± 10.5***	2.6 ± 1.9	4.4 ± 1.6	17.4 ± 2.7***	1.5 ± 0.5	3.7 ± 0.9	21.4 ± 5.3***
	Macrophages (x10^3^)	56.4 ± 4.2	58.9 ± 8.5	57.3 ± 6.9	84.2 ± 0.8	56.5 ± 6.2	69.7 ± 6.3	128.9 ± 8.2***	69.2 ± 7.9	85.6 ± 5.6*	106.6 ± 6.8***
	Eosinophils (x10^3^)	0.4 ± 0.6	0.5 ± 0.2	0.9 ± 0.3	9.7 ± 6.5	4.8 ± 0.4	8.4 ± 2.2***	45.0 ± 8.3***	0.9 ± 0.3	7.9 ± 3.8***	19.0 ± 7.5***
	Lymphocytes (x10^3^)	0.9 ± 0.2	0.8 ± 0.3	0.8 ± 0.2	4.6 ± 1.8	1.5 ± 0.7	6.7 ± 1.6***	14.1 ± 4.4***	1.3 ± 0.3	2.9 ± 1.3	8.5 ± 3.4*
	Total BAL cells (x10^3^)	69.1 ± 6.4	66.9 ± 8.6	71.8 ± 8.3	185.3 ± 14.0***	69.3 ± 6.6	99.4 ± 7.6	221.4 ± 17.2***	81.0 ± 8.2	109.3 ± 4.8***	174.9 ± 19.4***
28 days											

	Neutrophils (x10^3^)	1.2 ± 0.2	1.1 ± 0.3	2.8 ± 0.6	14.7 ± 2.7***	0.6 ± 0.4	1.2 ± 0.4	1.4 ± 0.2	1.1 ± 0.4	1.8 ± 0.9	6.6 ± 2.0
	Macrophages (x10^3^)	82.4 ± 5.7	79.5 ± 5.4	71.8 ± 10.7	106.2 ± 14.6	54.1 ± 3.6	64.0 ± 3.6	91.2 ± 13.3	94.6 ± 23.5	92.8 ± 6.1	141.5 ± 30.4
	Eosinophils (x10^3^)	0.3 ± 0.0	0.5 ± 0.5	1.6 ± 0.0	1.2 ± 1.1	0.0 ± 0.0	1.9 ± 1.5	0.1 ± 0.1	0.07 ± 0.07	0.7 ± 0.6	3.2 ± 2.9
	Lymphocytes (x10^3^)	2.1 ± 0.4	2.0 ± 0.4	3.8 ± 1.0	18.4 ± 3.8	1.4 ± 0.4	2.8 ± 0.6	5.0 ± 1.3	2.0 ± 1.0	1.6 ± 0.5	5.8 ± 2.4
	Total BAL cells (x10^3^)	95.9 ± 5.8	94.1 ± 6.9	85.8 ± 11.6	154.7 ± 16.0*	62.5 ± 3.9	79.5 ± 4.9	108.8 ± 13.0	104.6 ± 25.0*	107.9 ± 6.0	173.6 ± 28.6**#

BAL fluid counts in mice. 1, 3 and 28 days post-exposure to 18 μg, 54 μg and 162 μg NanoTiO2, 54 μg, 162 μg and 486 μg sanding dusts and control mice. *, **, ***: Statistically significant compared to control mice at the 0.5, 0.01 and 0.001 level, respectively. #: Statistically significant difference between the two paints at the 0.05 level

The total number of BAL cells was not significantly higher in mice exposed to 18 μg of NanoTiO_2 _and 54 μg of sanding dusts at any time point, compared with the negative control group (Table 2). In mice exposed to 54 μg of NanoTiO_2 _, the total number of BAL cells was only higher 1 day after intratracheal instillation, whereas the total number of BAL cells was significantly higher at all-time points in mice exposed to 162 μg of NanoTiO_2 _.

In mice exposed to 162 μg of Indoor-NanoTiO_2_, the total number of BAL cells was significantly higher 1 day and 3 days after intratracheal instillation, while this was still present 28 days after instillation in mice exposed to 486 μg of Indoor-NanoTiO_2 _dust.

In mice exposed to 162 μg of Indoor-R dust, the total number of BAL cells was significantly higher 1 day after intratracheal instillation, while this increase was still present 3 days after intratracheal instillation in mice exposed to 486 μg of Indoor-R dust.

The neutrophil count was not significantly higher in mice exposed to 18 μg of NanoTiO_2 _at any time point, compared with the negative control group (Figure 1, Table 2). In mice exposed to 54 μg of NanoTiO_2_, the neutrophil count was only significantly higher 1 and 3 days after intratracheal instillation, whereas the neutrophil count was significantly higher at all-time points in mice exposed to 162 μg of NanoTiO_2 _(162 μg). However, the increase was moderate after 28 days. As described in [19], neutrophil counts were higher at all doses of Printex 90 1 and 3 days after intratracheal instillation, while only 54 and 162 μg of Printex 90 resulted in increased neutrophil influx 28 days after intratracheal instillation. Sanding dust from both paints resulted in increased neutrophil counts in mice 1 day after intratracheal instillation of 162 and 486 μg. Exposure to 486 μg of both types of sanding dust resulted in increased neutrophil numbers 3 days after intratracheal instillation, while no increased influx of neutrophils were observed 28 days after intratracheal instillation. The neutrophil counts were not different between the NanoTiO_2 _containing paint (Indoor-NanoTiO_2_) and the reference paint without NanoTiO_2 _(Indoor-R). Thus, inflammation was observed for all tested materials, although the paint dust with and without nanoparticles was much less inflammogenic than the pure NanoTiO_2 _at similar mass. One day after intratracheal instillation, 162 μg of sanding dust was lowest observed adverse effect level (LOAEL) whereas 54 μg was no observed adverse effect level (NOAEL). The corresponding values for instillation of NanoTiO_2 _were 54 μg (LOAEL) and 18 μg (NOAEL).

Significantly higher numbers of macrophages were only seen 3 days after intratracheal instillation in mice exposed to Indoor-NanoTiO_2 _(162 and 486 μg) and Indoor-R (486 μg) (Table 2).

Higher numbers of eosinophils were observed in mice instilled with Indoor-NanoTiO_2 _and Indoor-R 1 day after intratracheal instillation of 486 μg and 3 days after intratracheal instillation of 162 and 486 μg. No eosinophils were detected at any time-point in mice exposed to NanoTiO_2_. Increased numbers of lymphocytes were detected 3 days after intratracheal instillation in mice exposed to 486 μg of Indoor-NanoTiO_2 _and 162 and 486 μg of Indoor-R (Table 2).

The only significant difference between the two paints was an increase (~1.6-fold) in the total number of BAL cells in the Indoor-NanoTiO_2 _exposed mice compared to Indoor-R exposed mice (Table 2).

### Tgf-β mRNA expression in the lungs

The pulmonary Tgf-*β *mRNA expression levels were assessed as a marker for a profibrotic response. The Tgf-*β *mRNA expression levels were not affected by any of the tested materials (all doses at all-time points) compared to control mice (results not shown).

### DNA damage

**Figure 2:**
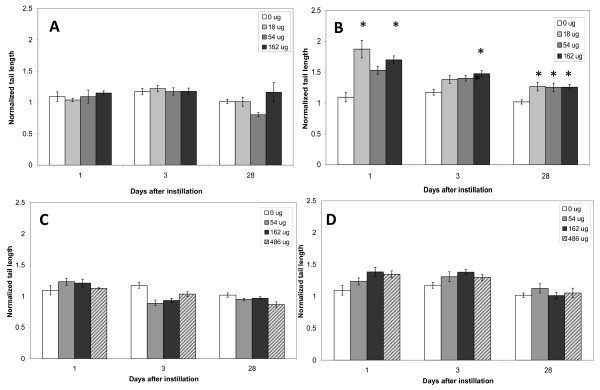
**DNA strand breaks in BAL cells**. DNA strand breaks in BAL cells obtained from mice exposed to 0, 18, 54 or 162 μg of NanoTiO_2 _(A) or Printex 90 (B) or 0, 54, 162 or 486 μg of Indoor-R (C) or Indoor-NanoTiO_2 _(D). The statistical analysis for Printex 90 has been described previously [19]. *, **, ***: Statistically significant compared to control mice at the 0.5, 0.01 and 0.001 level, respectively.

**Figure 3:**
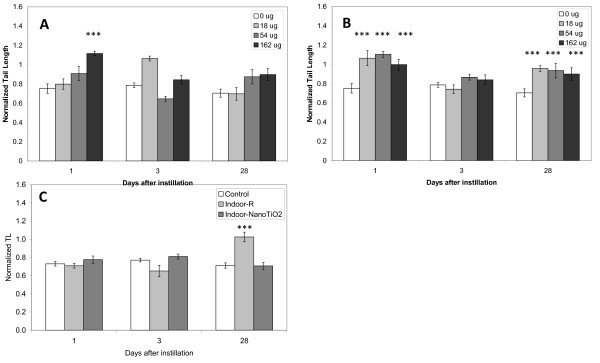
**DNA strand breaks in hepatic tissue**. DNA strand breaks in liver tissue from mice exposed to 0, 18, 54 or 162 μg of NanoTiO_2 _(A) or Printex 90 (B) or 0 or 486 μg of Indoor-R or Indoor-NanoTiO_2 _(C). The statistical analysis for Printex 90 has been described previously [19]. The increased level of DNA strand breaks of Indoor-R is considered an artifact due to 1) a low plate control and the normalization procedure and 2) the unusual kinetic of DNA damage over time. *, **, ***: Statistically significant compared to control mice at the 0.5, 0.01 and 0.001 level, respectively.

DNA damage was determined in BAL cells (Figure 2) and liver tissue (Figure 3) by the Comet assay.

#### BAL cells

Neither the NanoTiO_2 _nor Indoor-TiO_2 _and Indoor-R resulted in significant increases in DNA strand breaks in BAL cells at any of the tested doses and time points. Printex 90 induced a statistically significant increase in DNA strand breaks in the BAL cells 1 day after intratracheal instillation of 18 and 162 μg and 28 days after intratracheal instillation of all doses (18, 54, 162 μg), while there was only effect at the 162 μg dose 3 days after intratracheal instillation [19].

#### Liver tissue

Because only Printex 90 induced DNA strand breaks in BAL cells, initially we analysed the DNA damaging effect of the four materials only at the highest dose in liver (162 μg for NanoTiO_2 _and Printex 90; 486 μg for Indoor-NanoTiO_2 _and Indoor-R) at all time points. There was more DNA damage in hepatic tissue from mice exposed to Printex 90 and NanoTiO_2 _than in tissue from mice exposed to Indoor-NanoTiO_2 _and Indoor-R even though much higher doses were used for the paint dusts. Therefore the DNA damaging effects were examined at all doses and all times only after NanoTiO_2 _and Printex 90 exposures. Intratracheal instillation of 162 μg of NanoTiO_2 _resulted in increased level of strand breaks in the liver 1 day after exposure, while this effect disappeared at the later time points (Figure 3.A). Printex 90 induced higher levels of DNA damage at all doses 1 and 28 days after intratracheal instillation, while there was no effect 3 days after intratracheal instillation (Figure 3.B) [19].

For the mice exposed to the two types of dusts, Indoor-R resulted in significant increased level of DNA strand breaks only on day 28 (Figure 3.C). However, we believe this was an artefact in the normalization due to a low plate control.

### Liver histology

**Figure 4:**
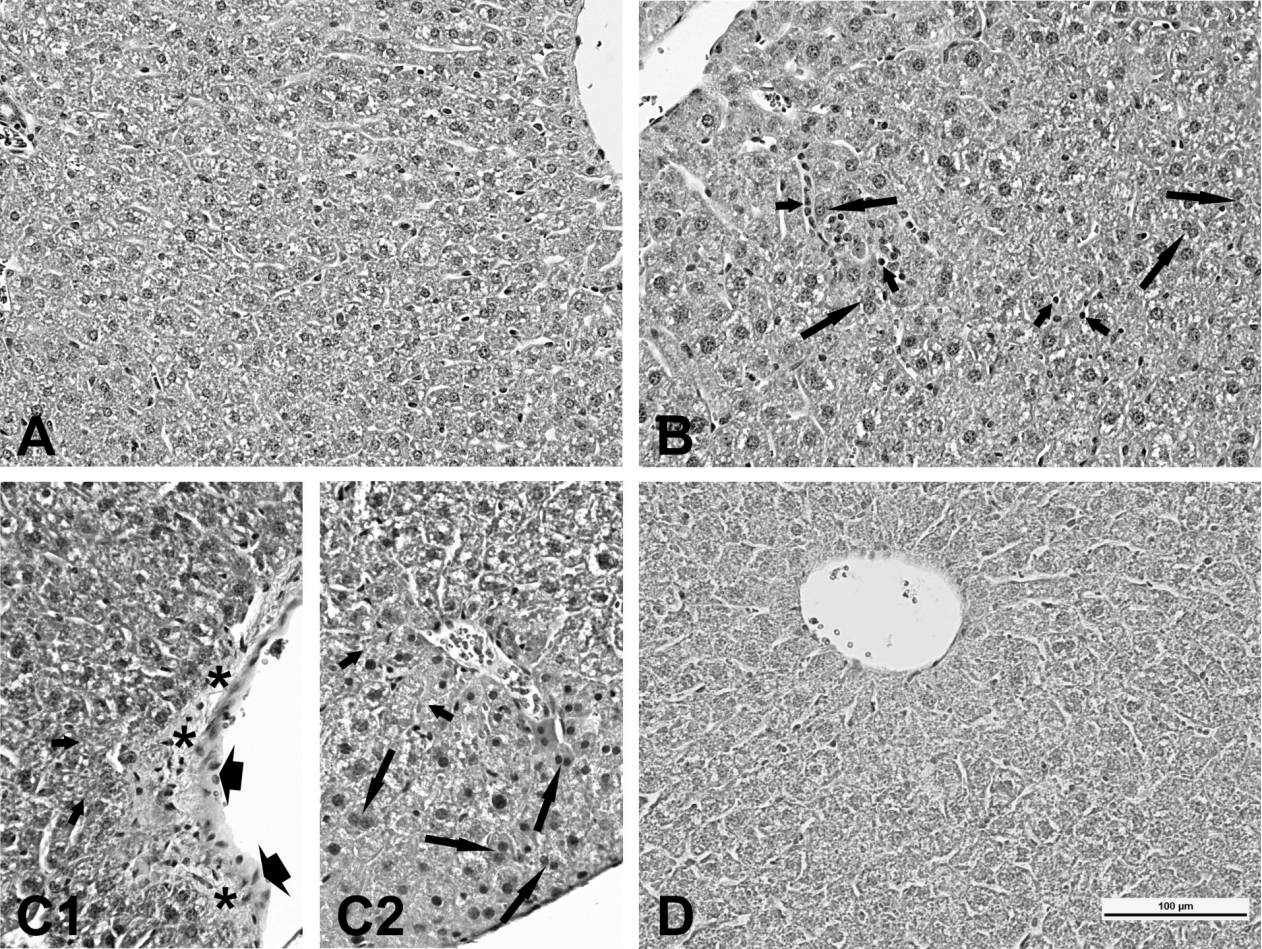
**Liver histology**. Microscopic pattern of the liver mice: A (vehicle exposed control animals) - normal structure; B (mice exposed to NanoTiO_2 _- 24 h after exposure) - infiltration of inflammatory cells (short arrows), binucleate hepatocytes (long arrows); C (mice exposed to Printex 90 - 28 days after exposure) - microfoci of necrosis (short arrows), C1 - edematous of endothelial cells (head of arrows), hyperplasia of connective tissue (asterisks), C2 - binucleate hepatocytes (long arrows); D (dust from Indoor-NanoTiO_2 _- 3 days after exposure) - normal structure; staining HE, magnification as scale on the figure D.

As compared to the controls histopathological lesions were observed in livers from mice exposed to 162 μg of NanoTiO_2 _or Printex 90 at different time points (primarily at day 28) (Table 3 and Figure 4). All histopathological changes were slight in severity. No histopathological changes were recorded in livers from mice exposed to either 486 μg of Indoor-NanoTiO_2 _or Indoor-R at any time point examined.

**Table 3:** Type and incidence of histopathological lesions.

Type of lesion/post-treatment day	Control	Nano TiO_2_	Indoor-Nano TiO_2_	Indoor-R	Printex 90
Foci (small) of inflammatory cells					
Day 1	0/3^a^	**1/4**	0/6	0/6	0/4
Day 3	0/3	0/4	0/5	0/6	0/4
Day 28	0/5	**1/5**	0/5	0/5	0/6

Polymorphonuclear cell foci					
Day 1	0/3	0/4	0/6	0/6	**1/4**
Day 3	0/3	0/4	0/5	0/6	**2/4**
Day 28	0/5	0/5	0/5	0/5	**2/6**

Hyperplasia of connective tissue perivascular					
Day 1	0/3	**1/4**	0/6	0/6	0/4
Day 3	0/3	**1/4**	0/5	0/6	0/4
Day 28	0/5	**1/5**	0/5	0/5	0/6

Hyperplasia of connective tissue near bile ductules or venules					
Day 1	0/3	0/4	0/6	0/5	0/4
Day 3	0/3	0/4	0/5	0/6	0/4
Day 28	0/5	0/5	0/5	0/5	**3/6**

Necrosis in centrilobular area					
Day 1	0/3	0/4	0/6	0/6	0/4
Day 3	0/3	0/4	0/5	0/6	0/4
Day 28	0/5	**3/5**	0/5	0/5	0/6

Microfoci of necrosis in centrilobular area					
Day 1	0/3	0/4	0/6	0/6	0/4
Day 3	0/3	0/4	0/5	0/6	0/4
Day 28	0/5	0/5	0/5	0/5	**1/6**

Eosinophilic necrosis in single hepatocytes					
Day 1	0/3	0/4	0/6	0/6	0/4
Day 3	0/3	0/4	0/5	0/6	0/4
Day 28	0/5	0/5	0/5	0/5	**1/6**

Hepatocytes with pycnotic nuclei					
Day 1	0/3	0/4	0/6	0/6	**1/4**
Day 3	0/3	0/4	0/5	0/6	**1/4**
Day 28	0/5	0/5	0/5	0/5	**1/6**

Parenchymal degeneration					
Day 1	0/3	0/4	0/6	0/6	0/4
Day 3	0/3	0/4	0/5	0/6	0/4
Day 28	0/5	**2/5**	0/5	0/5	**3/6**

Binucleate hepatocytes					
Day 1	0/3	**1/4**	0/6	0/5	0/4
Day 3	0/3	0/4	0/5	0/6	0/4
Day 28	0/5	0/5	0/5	0/5	**2/6**

Oedema of endothelial cells of portal venules					
Day 1	0/3	0/4	0/5	0/6	0/4
Day 3	0/3	0/4	0/5	0/6	0/4
Day 28	0/5	0/5	0/6	0/5	**2/6**

Increased number of Kupffer cells^b^					
Day 1	0/3	**2/4**	0/6	0/6	**1/4**
Day 3	0/3	**2/4**	0/5	0/6	**1/4**
Day 28	0/5	**2/5**	0/5	0/5	**2/6**

Type and incidence of histopathological lesions in the control mice and mice exposed to 162 μg of NanoTiO_2_, 486 μg Indoor-NanoTiO_2_, 486 μg Indoor-R, or 162 μg of Printex 90. ^a^: Incidence of a lesion expressed as a number of livers with the lesion of a total number of examined livers. ^b^: Qualitative evaluation only.

## Discussion

In the present study, we investigated the dose-response relations of inflammation and DNA damage of one titanium dioxide nanomaterial (UV-Titan L181, code: NanoTiO_2_) and sanding dust from paints with (Indoor-NanoTiO_2_) and without NanoTiO_2 _(Indoor-R) 1, 3 and 28 days after a single intratracheal instillation in mice. Our results show that there was no additive effect of adding NanoTiO_2 _to the paint compared to the reference paint for any of the measured toxicological endpoints. Furthermore the results show that when evaluated on mass-basis, NanoTiO_2 _elicited greater pulmonary inflammation than the sanding dusts. NanoTiO_2 _caused DNA damage in liver tissue 1 day after intratracheal instillation, while no DNA damaging effects were observed in BAL cells at any of the measured time points. The sanding dusts caused neither DNA damage in the liver at the highest dose nor in the BAL cells at any dose except for the increased level of DNA damage in the liver from the Indoor-R exposed mice at day 28. However, we consider this finding likely to be a chance finding due to a low plate control used in the normalization procedure and due to the unusual kinetic of DNA damage (only DNA damage after 28 days). No histopathological lesions were detected in paint dust exposed mice, whereas modest histopathological changes were detected in livers of mice treated with the pure nanomaterials.

### Selection of test materials and study design

The tested materials were chosen based on results from our two recent publications, in which we investigated the inflammatory and genotoxic effects in mice 1 day after a single dose (54 μg) of 1) a panel of nanoparticles with potential use in paints and lacquers [14] and 2) sanding dusts from a panel of paints with and without nanoparticles [12]. We found that despite that several of the nanoparticles were inflammogenic, the sanding dusts from nanoparticle containing paints did not result in an increased toxicological response compared to sanding dusts from reference paints. Based on these previous studies, we selected one TiO_2 _nanomaterial (NanoTiO_2_) and sanding dusts from the corresponding paints with (Indoor-NanoTiO_2_) and without NanoTiO_2 _(Indoor-R) to be tested for dose-responses at different times because NanoTiO_2 _was inflammogenic and genotoxic [14]. The tested nanopaint contained 10% NanoTiO_2_. To be able to compare the same amount of NanoTiO_2 _in paint we chose two different dose ranges for the pure NanoTiO_2 _(18, 54, 162 μg) and the sanding dusts (54, 162, 486 μg). The 18 μg NanoTiO_2 _dose approximately corresponds to the NanoTiO_2 _content in a 162 μg of sanding dust and similarly for the 54 μg dose of NanoTiO_2 _and 486 μg dose of sanding dust (Indoor-NanoTiO_2_). The carbon black type Printex 90 was included as a positive control because we have previously shown that Printex 90 is a potent ROS producer [15] that causes both DNA strand breaks and mutations [16].

### Dose considerations

The NanoTiO_2 _doses (18, 54 and 162 μg) equal 1.5, 5 and 15 working days at the Danish occupational exposure level for TiO_2 _(6.0 mg Ti/m^3 ^~ 9.75 mg TiO_2_/m^3^) (assuming that 9% of the inhaled mass ends up in the pulmonary region [20], volume of inhaled air per hour 1.8 l/h [21] and 8 hour working days).

### Toxicity of NanoTiO2 when bound in a paint matrix

Our previous single dose study of a range of different paint dusts in mice 1 day after intratracheal instillation showed that the majority of nanoparticles seem to stay in the paint matrix after sanding [12]. This is also the general view that exposure to nanosized titanium dioxide occurring during use of products is insignificant when the titanium dioxide is bound to other materials, such as in paints [22]. In the present study, we compared the dose-response relations at several time points of NanoTiO_2 _as part of a paint matrix with the effects of pure NanoTiO_2_. By choosing the different dose ranges for NanoTiO_2 _and sanding dusts, the study was designed to investigate whether it was possible to detect an additive effect or synergetic effect of the NanoTiO_2 _in the Indoor-NanoTiO_2 _compared to the Indoor-R. However, no increase in any of the toxicological endpoints was detected for the Indoor-NanoTiO_2 _compared to the Indoor-R. SEM analysis of the Indoor-NanoTiO_2 _suspension used for instillation indicated that most of the NanoTiO_2 _apparently was retained in the paint matrix [12]. Immediately, the results suggest that the inflammogenic effects of NanoTiO_2 _are masked when incorporated into a matrix and this may explain why we observed the same inflammatory response for both paints. If an additive effect was expected (e.g, all included NanoTiO_2 _was bio accessible), we would have observed a 20% increase in the number of neutrophils at day 1.

The instilled dose of Indoor-NanoTiO_2 _can be viewed as the combination of the middle dose of NanoTiO_2 _(54 μg) and the highest dose of Indoor-R (486 μg). We observed induction of liver DNA damage only at the highest dose of NanoTiO_2 _and not at the high dose of Indoor-NanoTiO_2_. The induction of liver DNA damage by Indoor-R we think was a chance finding. Thus, taking these results into account, we did not expect that exposure to Indoor-NanoTiO_2 _at the chosen dose would lead to genotoxicity and the results confirm this. Because similar toxicological responses for the measured endpoints were observed for the nanoparticle containing paint compared to the conventional paint, our data support our previous findings showing that nanomaterials bound in a paint dust do not contribute to the observed toxicity [12]. Therefore, our results support a very recent publication by Wohlleben et al. investigating the inflammatory and genotoxic response of nanomaterials and sanding dusts from cement and plastic composites with and without nanomaterial after intratracheal instillation in rats [6]. In that study, similar to our study no additional toxicity was observed for the nanomaterial containing dusts compared to dusts from reference products.

### Pulmonary inflammation

For all the tested materials the greatest number of neutrophils was observed 1 day after exposure. Mice exposed to the highest dose of NanoTiO_2 _(162 μg) still had increased numbers of neutrophils 28 days after exposure. This is in line with our previous publication showing increased inflammation in mice 4 weeks after end of exposure to the same NanoTiO_2 _by inhalation to 42 mg/m^3 ^aerolized powder for 1h/day on 11 consecutive days [20]. In that study, 21% of the deposited NanoTiO_2 _could be detected in lung tissue 28 days after last exposure. The neutrophil influx in mice exposed to the two different sanding dusts were similar to each other and of much smaller scale than the inflammation in mice exposed to nanomaterials when comparing the mass dose.

**Figure 5:**
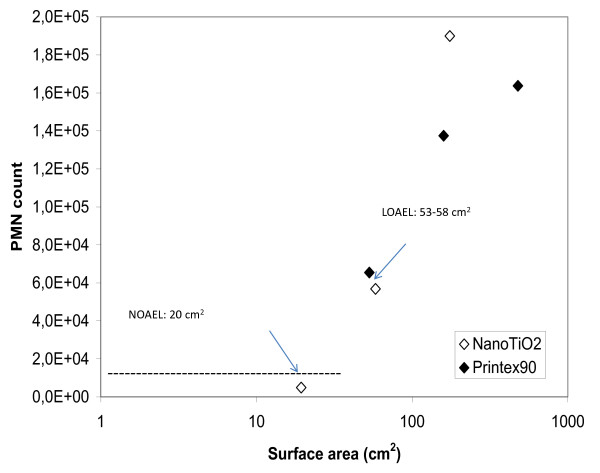
**Correlation between surface area and neutrophil influx**. The threshold for inflammatory response for nanoparticles is shown as the correlation between instilled particle surface area and neutrophil cell numbers. The dashed line indicates baseline-level of inflammation in control mice. NOAEL: No observed adverse effects level LOAEL: Lowest observed adverse effect level.

There is much evidence that the inflammatory response induced by low-toxicity low-solubility particles correlates well with the instilled surface area of the particles [14,23-26]. We found that the specific surface area of NanoTiO_2 _and Printex 90 correlated strongly with the influx of neutrophils (Figure 5). The results show that the NOEL is 19 cm^2 ^and the LOEL is 53 and 58 cm^2 ^(Printex 90 and NanoTiO_2_, respectively). Our results are in line with a previous study by Stöger and colleagues demonstrating the existence of a threshold for the particle surface area at an instilled dose of approximately 20 cm^2^, below which no inflammatory responses could be detected in mice 1 day after intratracheal instillation [27]. In the present study, the effects were reversible over time. The neutrophilic response declined over time from day 1 to 3 and to 28 days after intratracheal instillation. BET analysis was not possible for the paint dust particles due to insufficient amounts of materials. However, estimations from the total airborne particle surface areas and volumes in Koponen et al. [7], suggest that the volume specific surface areas of the sanding dust particles were in the order of 2.8 to 3.5 m^2^/cm^3^. The specific surface area of NanoTiO_2 _and Printex 90 were 107.7 and 295 m^2^/g, respectively. Hence, assuming a density of 1 g/ml, the instilled paint surface area will only be on the order of 1 to 3% of the specific surface area for the same dose of the pure nanomaterials. This suggests that other factors such as particle chemistry are also important for the toxicity.

### Pulmonary fibrosis

Some nanomaterials have been shown to induce fibrosis as e.g. CNTs [17]. The pulmonary *Tgf-β *mRNA expression level was assessed as a marker for a profibrotic response. The level of TGF-β in BAL fluid was increased in mice exposed to CNTs after exposure by apiration [28] and by intratracheal instillation [29]. However, in the present study the *Tgf-β *mRNA expression levels were not affected by exposure to any of the tested materials (all doses at all time points) compared to control mice. It is possible that we by measuring *Tgf-β *mRNA expression levels at 1, 3 and 28 days after intratracheal instillation missed the optimal time frame for assessing TGF-β. In both the above mentioned studies a maximum for the TGF-β level in BAL fluid was found 7 days after exposure. However, another explanation for the differences could be that the tested materials in the present study do not have the same fibrous nature as CNTs. Our results are in line with a recent study of markers of inflammation and fibrosis in mice where no increase in TGF-β1 level in BAL fluid was found 2, 7, 14 and 21 days after intratracheal instillation of 10 μg Printex 90 [30]. It should be noted that fibrosis is a very complex pathway. We chose to evaluate *Tgf-β *mRNA expression as a marker of fibrosis because this marker has been used previously with success within the area of nanotoxicology [28]. However, a panel of markers for tissue fibrosis exists [31] and it might be that other markers of fibrosis would have been informative as well.

### DNA damage in BAL cells

In BAL cells, except for the reference particle Printex 90 [19] none of the tested products induced DNA damage by the comet assay. So the data suggest that the products do not cause DNA damage at the time of analysis, when evaluated by the Comet assay. The reference particle Printex 90 caused DNA damage in BAL cells 1 day after intratracheal instillation at all tested doses. We have previously shown that exposure to Printex 90 resulted in DNA damage 3 hours after pulmonary exposure in ApoE-/- mice [26]. DNA strand breaks last only for a short-term, probably because the DNA damage is rapidly repaired by DNA repair enzymes [32].

### Hepatic effects

In the liver, NanoTiO_2 _induced DNA damage 1 day after intratracheal instillation at the highest dose tested, while no effect was seen in sanding dust exposed mice. The reference particle Printex 90 caused a biphasic response for the DNA damage [19].

Exposure of mice to the sanding dust from paints with and without NanoTiO_2 _was not associated with any hepatic histopathological changes compared to the vehicle controls. In contrast, exposure to NanoTiO_2 _and to Printex 90 was associated with modest histopathological changes in the liver primarily at day 28 (Table 3, Figure 4).

An increased number of binucleate hepatocyte can be indicative of a regenerative activity in the tissue exposed to a toxic agent [33]. Changes such as inflammatory cell foci, polymorphonuclear cell foci, or microfoci of necrosis are known to occur also under physiological conditions in untreated animals [34] and binucleate hepatocytes are often noted in the aged mice [34]. On the other hand, the animals used in the present study were 12 weeks at the latest time point and no changes were observed in the control mice. The hepatic effects in terms of DNA damage and modest histological changes seen in the present study can hypothetically be caused both by systemic inflammation and by direct effects of translocated particles. That we detected the largest effects with the smallest particles (NanoTiO_2 _and Printex 90) support an interpretation that the observed hepatic effects may indeed be caused by nanoparticle exposure because: 1) NanoTiO_2_/Printex90 caused more persistent inflammation over time than the sanding dusts, and 2) the translocation of NanoTiO_2_/Printex90 would be expected to be larger than the sanding dusts due to their smaller size. Hepatic DNA damage following Printex 90 exposure was reported in our recent publication showing DNA strand breaks in time-mated mice exposed by inhalation to 42 mg/m^3 ^Printex 90 for 1 h/day on gestation days (GD) 8-18 mice 5 and 24 days after inhalation exposure, while no effect was found after intratracheal instillation of corresponding pulmonary dose at similar time-points [35]. Histopathological changes such as cytoplasmic vacuolisation and hepatic focal necrosis or minimal to moderate bile duct hyperplasia, single cell necrosis (characterized by increased cellular eosinophilia and shrunken condensed nuclei), and moderate multifocal necrosis were reported in some of the rats exposed by inhalation to either 19 nm silver nanoparticles (6 h/day, 5 days/week, for 13 weeks) [36] or to silver nanoparticles sized 2-65 nm (6 h/day, 5 days/week, for 4 weeks) [37].

## Conclusions

NanoTiO_2 _did not induce DNA damage in lung lining cells despite being highly inflammogenic. In contrast, NanoTiO_2 _induced hepatic DNA damage at the highest tested dose. Sanding dusts from both paints resulted in increased pulmonary inflammation while no DNA damage was observed in BAL cells or liver tissue. The level of pulmonary inflammation in mice exposed to sanding dust was not affected by the addition of nanoparticles to paint.

## Methods

### Animals

Female C57BL/6 mice 5-7 weeks old were obtained from Taconic (Ry, Denmark). The mice were allowed to acclimatize for 1-3 weeks before the experiment. All mice were given food (Altromin no. 1324, Christian Petersen, Denmark) and water *ad libitum *during the whole experiment. The mice were group housed in polypropylene cages with sawdust bedding and enrichment at controlled temperature 21 ± 1°C and humidity 50 ± 10% with a 12-h light:12-h dark cycle. Female mice were studied at 8 weeks of age. The experiments were approved by the Danish "Animal Experiments Inspectorate" and carried out following their guidelines for ethical conduct and care when using animals in research.

### Particles and sanding dusts

#### Products

The Danish paint and lacquer industry provided an indoor acrylic paint product with 10% content of NanoTiO_2 _(referred as Indoor-NanoTiO_2_) and a corresponding product without nanoparticles (referred to as Indoor R) (Table 1). The tested pure nanomaterials comprised of a nanosized TiO_2 _material (UV-Titan L181, code: NanoTiO_2_), and carbon black (Printex 90), which was included as an internal reference particle. Printex 90 was a gift from Degüssa (Germany). As reported previously, the specific surface areas of the particles were 295 m^2^/g for Printex 90 [38] and 107.7 m^2^/g for NanoTiO2 [14]. The nanoparticles and the paints are described in detail in [12] and [14].

#### Generation of dusts

Dusts were generated by sanding of boards painted with the two selected paints paints as described previously [7]. Briefly, dust samples were collected by an electrostatic precipitor. The device was modified from a commercial air cleaner and is described in details in [18]. Paint dust was deposited on the silver coated plates. After sampling the plate holder system was covered with Aluminum-foil and carefully transported to the laboratory where dust was removed from the plates using a silicon scraper. The samples were stored in the freezer until toxicological testing.

### Preparation of exposure stock

Particles were suspended by sonication in 0.9% NaCl MilliQ water containing 10% v/v acellular BAL collected from C57BL/6 mice. The BAL fluid was prepared by flushing unexposed mice twice to 0.6 ml 0.9% NaCl yielding approximately 1 ml of BAL fluid. Acellular BAL was prepared by centrifugation of BAL fluid at 400 g (10 min, 4°C). The particles (4.05 mg/ml) and dust suspensions (12.15 mg/ml) were sonicated using a Branson Sonifier S-450D (Branson Ultrasonics Corp., Danbury, CT, USA) equipped with a disruptor horn (Model number: 101-147-037). Total sonication time was 16 min, with alternating 10 s pulses and 10 s pauses at amplitude of 10% (8 min sonication total). During the sonication procedure the samples were continuously cooled on ice. These suspensions were used for the high dose (486 μg (dust) and 162 μg (NanoTiO_2_/Printex 90)) and diluted 1:3 for the medium dose and diluted further 1:3 for the low dose. Between the dilutions the suspensions were pipetted. Vehicle control solutions were prepared containing 90% 0.9% NaCl MilliQ water and 10% acellular BAL fluid.

### Characterization of exposure

Characterization of the materials in instillation vehicle have been published previously [12,14]. Briefly, the average size of the materials in instillation vehicle were determined by Dynamic Light Scattering (DLS) and the shapes of the materials and the extent of agglomeration/aggregation in instillation vehicle were characterized by transmission electron microscopy (TEM) and scanning electron microscopy (SEM). TEM pictures of the NanoTiO_2 _suspension used for instillation showed that the individual nanoparticles mainly occurred in partially open to dense aggregates of ca. 100 nm size or larger and the size-distribution plot by DLS indicated a multimodal size-distribution with two modes in the μm-range (peaks at 5.5 and 2.3 μm) and one in the sub-μm range (825 nm) [14]. SEM analysis of the Indoor-NanoTiO_2 _and Indoor-R suspensions used for instillation suggests that most of the nanoparticles were still fully or partially encapsulated in the paint matrix after sanding. However, a considerable amount of the pigment particles were generally liberated from the matrix. Due to the size of the coarse dust fraction and sedimentation, DLS analysis was not reliable for the un-filtered dispersions of dusts [12].

### Measurement of endotoxin

An amount of 4.05 mg/ml of each type of nanoparticle and dust was suspended in pyrogen free water with 0.05% Tween 20 by orbital shaking (300 rpm) at room temperature. The particles were suspended by sonification as described above. The suspensions were centrifuged (20.000 rpm) for 6 min, and the supernatant was used for endotoxin assay. The endotoxin contents were analysed in duplicate using the kinetic *Limulus Amebocyte *Lysate test (Kinetic-QCL endotoxin kit, Lonza, Walkersville, MD, USA). A standard curve (ranging from 0.05 to 50 EU/ml) obtained from an Escherichia coli O55:B5 reference endotoxin was used to determine the concentrations in terms of endotoxin units (EU) (15.0 EU = 1 ng). In addition inhibition/enhancement controls were prepared by spiking supernatants from sample Indoor-NanoTiO_2_, Indoor-R, TiO_2 _and Printex 90 with 10 μl of a 0.5 EU/ml solution of endotoxin. As a control of the method NanoTiO_2 _and Printex 90 were tested twice (on two plates) and less than 16% variance was found.

### Exposure of mice

The mice were treated with a single intratracheal instillation with 18, 54 and 162 μg for the nanoparticles and 54, 162 and 486 for the paint dusts. To eliminate day to day variation, 3-4 materials including vehicle controls were instilled on each exposure day and each material dose was instilled on three separate days. Before the intratracheal instillation, the mice were anesthetized using Hypnorm^® ^(fentanyl citrate 0.315 mg/ml and fluanisone 10 mg/ml from Janssen Pharma) and Dormicum^® ^(Midazolam 5 mg/mL from Roche). Both were mixed with equal volume sterile water. A volume of 0.2 ml was injected subcutaneously into the loose skin over the neck of each mouse. The sedated mice were kept on 37°C heating plates. During instillation the mice were placed on their backs on a 40 degree slope. A one diode cold light source was placed touching the larynx. The tongue was pressed towards the lower jaw by a small spatula. The trachea was intubated using a 24 gauge BD Insyte catheter (Ref: 381212, Becton Dickinson, Denmark) with a shortened needle. The correct location of each intubation was tested by a small but highly sensitive pressure transducer (pneutachymeter), developed by our laboratory in collaboration with John Frederiksen (FFE/P, Copenhagen, Denmark). A 40 μl suspension was instilled followed by 150 μl air with a 250 μl SGE glass syringe (250F-LT-GT, MicroLab, Aarhus, Denmark). Control animals were instilled with vehicle (0.9% NaClwith 10% BAL). After the intubation catheter was removed, the mousebreathing was observed in order to assure that the delivered material did not block the airways. The mice were placed on to the 37°C heating plate until they recovered from anaesthesia.

### Preparation of tissue and cells from the mice

One, 3 or 28 days after intratracheal instillation, the mice were anaesthetised with Hypnorm/Dormicum as described above. Immediately after withdrawing the heart blood, a bronchoalveolar lavage (BAL) was performed four times with 0.8 mL of 0.9% sterile saline through the trachea. The BAL was immediately put on ice until BAL fluid and BAL cells were separated by centrifugation at 4°C and 400 g for 10 min. The BAL cells were resuspended in 100 μL medium (HAMF12 with 10% fetal bovine serum). Part of the suspension (40 μL) was mixed with 160 μL medium containing 10% DMSO and stored at -80°C for later analysis in the comet assay. For differential count, cells from 50 μL suspension were collected on microscope slides by centrifugation at 10,000 rpm for 4 min in a Cytofuge 2 (StatSpin, Bie and Berntsen, Rødovre, Denmark). The slides were fixed with 96% ethanol and stained with May-Grünwald-Giemsa stain. The cellular composition of BAL cells was determined on 200 cells. The total number of cells was determined by using the NucleoCounter (Chemometec, Allerød, Denmark) live/dead assay according to the manufacturer's instructions. The lungs and a piece of liver tissue were snap frozen in cryotubes (NUNC) in liquid N_2 _and stored at -80°C. From vehicle controls and high dose groups exposed to each of the test materials (3-6 per group) another piece of liver tissue from the left lobe was kept in formaldehyde (4%) until liver histology was performed.

### Preparation of RNA and cDNA from lung tissue

RNA was prepared using the NucleoSpin 96 RNA kit (Macherey-Nagel). RNA from the entire left lung of each mouse was prepared by lysing the tissue in 2 ml RLT buffer, while vigorously disrupting the sample with a Tissuelyser (Qiagen, Denmark) with a 5 mm stainless steel bead for 2 × 60 seconds and run through a QIAshredder (Qiagen, USA). The rest of the purification was performed as described by the manufacturer. cDNA was prepared using TaqMan reverse transcription reagents (Applied Biosystems, USA) as described by manufacturer.

### Real-time RT-PCR

The *Tgf-β1 *gene expression was determined using real-time RT-PCR with 18S RNA as reference gene (Kit nr. Mm03024053_m1 from Applied Biosystems). RT-PCR was performed using Universal Mastermix (Applied Biosystems, Nærum, Denmark). PCR was performed on an ABI PRISM^® ^7500 sequence detector (PE Biosystems, Foster City, CA, USA) as described previously [39]. 18S (4310893E, Applied Biosystems, Nærum, Denmark) was used as the reference gene. Expression for each gene was quantified in separate wells. Samples were quantified in triplicates; standard deviation was below 20%. Run to run variation was controlled by quantifying the mRNA levels for the same control sample. The standard deviation in separate runs was lower than 25%. No template and -RT controls were included in all runs.

### Comet assay

The level of DNA strand breaks in frozen BAL and liver tissue was determined by the alkaline comet assay as described in [40,41] based on a protocol by [42]. The strand breaks measured by the assay represent a mixture of direct strand breaks, alkaline labile sites and transient breaks in the DNA due to repair processes [43]. The samples were analyzed using a high throughput allowing 48 samples per GelBondfilm, as recently described [35] and (Gützkow et al: A high throughput comet assay using 96 A high throughput comet assay using 96 minigelsminigels, in preparation). BAL cell suspensions in freezing medium with 10% DMSO were thawed quickly. For liver, deep frozen samples (ca. 40 mg) were pressed through a metal stapler (diameter 0.5 cm, mesh size 0.4 mm) into Merchant's media (0.14 M NaCl, 1.47 mM KH_2_PO4, 2.7 mM KCl, 8.1 mM Na_2_HPO_4_, 10 mM NaEDTA, pH 7.4) for inhibiting endogenous DNA cleaving enzymes (first described by [44])). Samples were embedded in agarose, lysed, subjected to alkaline electrophoresis, fixed and later stained and scored as previously described [35]. Due to preparation time, the lysing procedure varied between 1-3.5 hour for samples in the present study. In order to minimize the effects of this we normalized the results to the positive assay control. As a positive assay control and to estimate the electrophoresis-to-electrophoresis variation, 0 and 30 μM H_2_O_2 _exposed A549 cells were included on each Gelbond film in all electrophoresis runs.

### Liver histology

Specimens were taken from the liver of three to six mice from the vehicle control from the high dose groups of all test materials and euthanized 1, 3 or 28 days after instillation. The specimens were fixed in 4% neutral buffered formaldehyde, paraffin-embedded, and sections 4-6 μm were made and stained with hematoxylin and eosin for histological examination.

### Statistics

The data were assessed by non-parametric three-way ANOVA with post-hoc Tukey-type multiple comparison test for effects showing statistical significance in the overall ANOVA test. Statistical significances were tested at P < 0.05 level. The statistical analyses were performed in SAS version 9.2 (SAS Institute Inc., Cary, NC, USA).

## Competing interests

The authors declare that they have no competing interests.

## Authors' contributions

HW, UV, NRJ and ATS were involved in the design of the study. IKK and KAJ generated the sanding dust. NRJ and ATS were involved in the design of the set-up of the mouse-exposure. AM and JS were responsible for the histological characterization. AMM carried out the endotoxin testing. HWA, NRJ, PJ, GB and KBG were responsible for the Comet assay. ATS was responsible for the toxicological data and submitted the manuscript. All authors approved the final manuscript.
